# Letter from the Editor in Chief

**DOI:** 10.19102/icrm.2023.14096

**Published:** 2023-09-15

**Authors:** Moussa Mansour


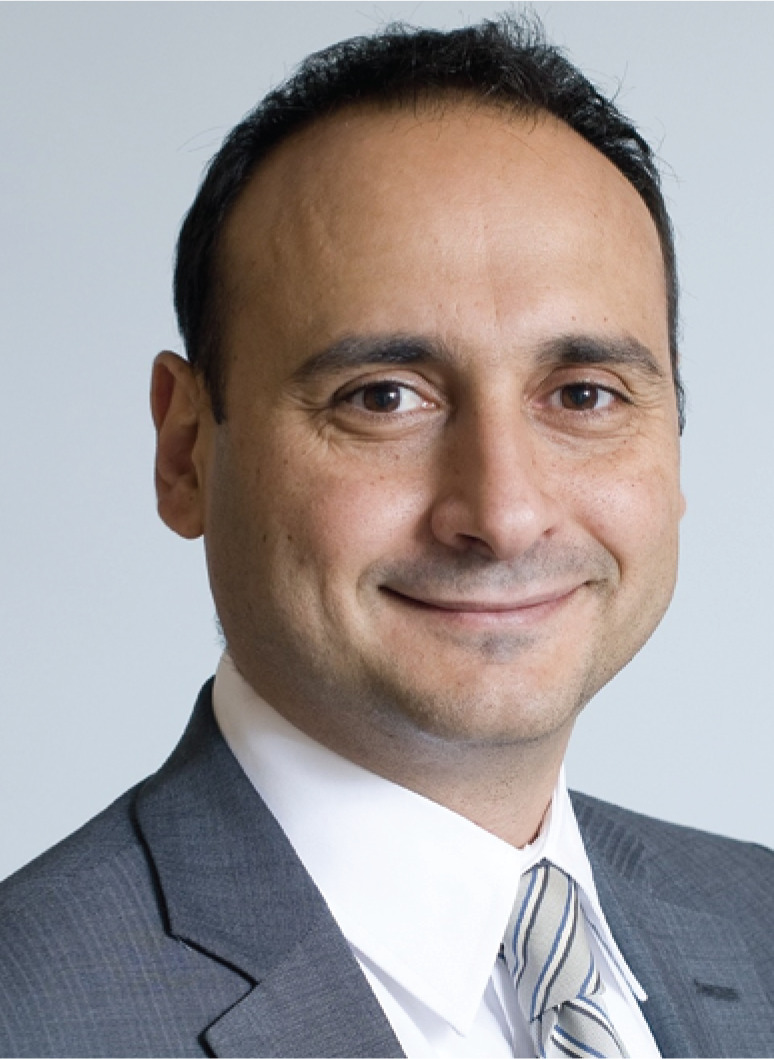


Dear readers,

The annual scientific meeting of the European Society of Cardiology took place in August in Amsterdam. Atrial fibrillation (AF) was well represented at the meeting, with many relevant studies presented during the late-breaking clinical trial sessions. I believe that two of these clinical trials will have significant impacts on the field of AF, and I highlight them here in this letter.

The first study is the Catheter Ablation for Atrial Fibrillation in Patients with End-stage Heart Failure and Eligibility for Heart Transplantation (CASTLE-HTx) trial,^1^ which enrolled patients with symptomatic AF and end-stage heart failure who were referred for heart transplantation evaluation. The subjects were randomized to receive either catheter ablation and guideline-directed medical therapy or medical therapy alone. The primary endpoint was a composite of death from any cause, implantation of a left ventricular assist device, or urgent heart transplantation. After a median follow-up of 18.0 (range, 14.6–22.6) months, the primary endpoint occurred in 8% of patients in the ablation group and 30% of patients in the medical-therapy group (hazard ratio, 0.24; 95% confidence interval [CI], 0.11–0.52; *P* < 0.001). Death from any cause occurred in 6% of patients in the ablation group and 20% of patients in the medical therapy group (hazard ratio, 0.29; 95% CI, 0.12–0.72). Procedure-related complications were minor. As a result, the combination of catheter ablation and guideline-directed medical therapy was associated with a reduced likelihood of a composite of death from any cause, implantation of a left ventricular assist device, or urgent heart transplantation when compared to medical therapy alone.

The second study of interest is the Randomized Controlled Trial for Pulsed Field Ablation versus Standard of Care Ablation for Paroxysmal Atrial Fibrillation (ADVENT) trial.^2^ This multicenter, prospective, single-blind, randomized controlled trial compared pulmonary vein isolation using pulsed-field ablation (PFA) with conventional thermal ablation for drug-resistant paroxysmal AF, with each site employing either (but not both) cryoballoon or radiofrequency ablation as a control condition. This was a non-inferiority study with a primary efficacy endpoint defined as freedom from a composite of initial procedural failure, documented atrial tachyarrhythmia after a 3-month blanking period, anti-arrhythmic drug use, cardioversion, or repeat ablation. The primary safety endpoint included acute and chronic device- and procedure-related serious adverse events. At 1 year, the primary efficacy endpoint was met (no events) in 73.3% of patients who underwent PFA and 71.3% of patients who underwent thermal ablation (between-group difference, 2.0 percentage points; 95% Bayesian credible interval, −5.2 to 9.2; posterior probability of non-inferiority, >0.999). Primary safety endpoint events occurred in 2.1% of patients who underwent PFA and 1.5% of those who underwent thermal ablation (between-group difference, 0.6 percentage points; 95% Bayesian credible interval, −1.5 to 2.8; posterior probability of non-inferiority, >0.999). As a result, ADVENT demonstrated that PFA was non-inferior to conventional thermal ablation in patients with paroxysmal AF receiving a catheter-based therapy with respect to freedom from a composite of initial procedural failure, documented atrial tachyarrhythmia, anti-arrhythmic drug use, cardioversion, or repeat ablation and with respect to device- and procedure-related serious adverse events at 1 year. Another important finding in ADVENT was that the procedure duration was significantly shorter with PFA.

These two studies are expected to mark the beginning of a new era in the field of catheter ablation for AF. CASTLE-HTx has the potential to expand the indications of catheter ablation for AF to a population of patients historically considered to have been too ill to benefit from ablation. Meanwhile, following ADVENT, PFA will likely become the primary technology used for pulmonary vein isolation. Interestingly, these studies also complement one another: AF ablation with PFA is quick and performed without fluid irrigation to cool the catheter, which are both features critical for patients with end-stage heart failure who cannot tolerate long procedures and fluid overload.

I hope that you enjoy reading the rest of this issue of *The Journal of Innovations in Cardiac Rhythm Management*.


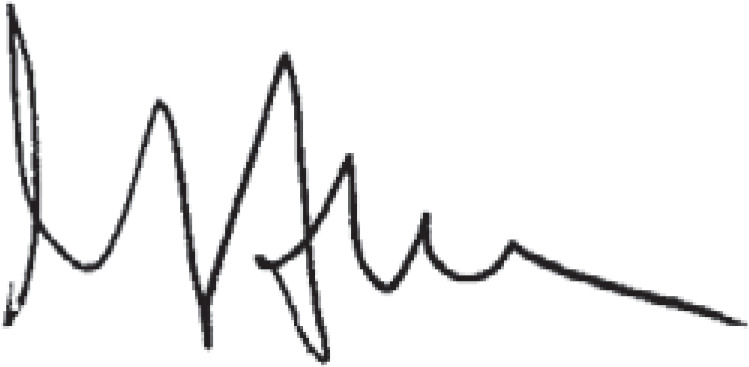


Sincerely,

Moussa Mansour, md, fhrs, facc

Editor in Chief

*The Journal of Innovations in Cardiac Rhythm Management*

MMansour@InnovationsInCRM.com

Director, Atrial Fibrillation Program

Jeremy Ruskin and Dan Starks Endowed Chair in Cardiology

Massachusetts General Hospital

Boston, MA 02114
